# dSir2 and Dmp53 interact to mediate aspects of CR-dependent life span extension in *D. melanogaster*

**DOI:** 10.18632/aging.100001

**Published:** 2008-11-06

**Authors:** Johannes H. Bauer, Siti Nur Sarah Morris, Chengyi Chang, Thomas Flatt*, Jason G. Wood, Stephen L. Helfand

**Affiliations:** Department of Molecular Biology, Cell Biology and Biochemistry, and *Department of Ecology and Evolutionary Biology, Division of Biology and Medicine, Brown University Providence, RI02912, USA

**Keywords:** Sir2, dSir2, p53, Dmp53, calorie restriction

## Abstract

Calorie Restriction (CR) is a well established method of extending life span in a variety of organisms. In the fruit fly *D. melanogaster*, CR is mediated at least in part by activation of dSir2. In mammalian systems, one of the critical targets of Sir2 is the tumor suppressor p53. This deacetylation of p53 by Sir2 leads to inhibition of p53's transcriptional activity. We have recently shown that inhibition of Dmp53 activity in the fly brain through the use of dominant-negative (DN) constructs that inhibit DNA-binding can extend life span. This life span extension appears to be related to CR, as CR and DN-Dmp53 do not display additive effects on life span. Here we report that life span extension by DN-Dmp53 expression is highly dynamic and can be achieved even when DN-Dmp53 is expressed later in life. In addition, we demonstrate that life span extension by activation of dSir2 and DN-Dmp53 expression are not additive. Furthermore, we show that dSir2 physically interacts with Dmp53 and can deacetylate Dmp53-derived peptides. Taken together, our data demonstrate that Dmp53 is a down stream target of dSir2 enzymatic activity and mediates some aspects of the life span extending effects of CR.

## Introduction

Calorie Restriction (CR) is an intervention with complex, pleiotropic effects that influence a plethora of biological processes. Animals on a CR regimen show decreased fecundity, increased activity and foraging behavior, and a general increase in stress resistance. Most importantly, CR consistently increases life span in most of the species tested. The mechanisms underlying these varied responses to a decrease in calorie intake remain unclear [1].

The fruit fly *D. melanogaster* has proven to be an invaluable tool in dissecting the molecular mechanisms of CR. Flies respond robustly with extended life spans when calories are limited. It has been suggested that it is the dilution of specific food constituents, i.e. yeast extract, that determines the life span of the fruit fly [2], as opposed to a reduction of total calories. However, the importance of this finding remains somewhat unclear [3], especially in light of the fact that yeast extract is a highly complex mixture of proteins, carbohydrates, salts, lipids, hormones and other small molecules [4].

Nonetheless, using food dilution methodologies of CR, several genes have been linked to CR. Life span extending mutations in the insulin receptor substrate CHICO [5] were shown to be non-additive to the life span extending effects of CR. Decreased rpd3 [6] and dSir2 over expression [7] both extend life span in a CR-related manner, corresponding to the observed down regulation of rpd3, and up regulation of dSir2 under CR conditions [6]. Most importantly, dSir2 null flies do not respond efficiently to CR [6]. Interestingly, the multitude of biological effects (fecundity, activity, etc.) of CR can be uncoupled from the life span extending effects of CR. dSir2 over expressing flies, while having extended life span, do not show any defects in fecundity. These data suggest that the life span extending effects of CR in flies are mediated at least in part by dSir2, while the effects on other physiological systems may branch off at points up stream of dSir2 in the CR signaling pathway [7].

We have recently identified the *Drosophila* homolog of the tumor suppressor p53 (Dmp53) as another candidate gene that might mediate the life span extending effects of CR. Expression of dominant-negative (DN) versions of Dmp53 in the adult fly brain extends life span, but is not additive to the effects of CR. The life span extending effects of DN-Dmp53 are furthermore smaller than the life span extending effects of CR. DN-Dmp53 long-lived flies show no decrease in fecundity or of physical activity, yet are resistant to oxidative stress. These data suggest that Dmp53 is one of the down stream elements of the CR signaling pathway [8].

Here we further examine the possible role of Dmp53 in the CR signaling pathway. Our results indicate that Dmp53 is part of the CR signaling mechanism. We show that DN-Dmp53- and dSir2-dependent life span extensions are not additive. Importantly, Dmp53 and dSir2 physically interact, suggesting that Dmp53 is a down stream target of dSir2 deacetylation activity. Our data provide for a molecular ordering of the known components of the CR pathway in fruit flies, and thus present a framework for future research and hypothesis testing.

## Results

### DN-Dmp53-dependent life span extension can be induced later in life and is reversible.

**Figure 1.**
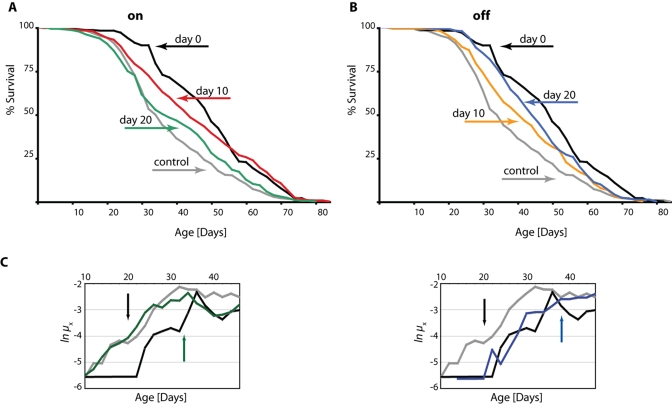
DN-Dmp53-dependent life span extension can be induced later in life and is reversible. Survivorship curves of female ELAV-Switch-DN-Dmp53 flies demonstrate plasticity. When DN-Dmp53 expression is turned on later in life (A; black: turned on at the day of eclosion; median life span 50 days; grey: always turned off; median life span 34 days; red: turned on at day 10; median life span 44 days; green: turned on at day 20; median life span 38 days) life span can still be increased, albeit to a lesser degree than in continuously expressing flies. Turning off DN-Dmp53 expression later in life leads to a shortening of life span extension (B; black: turned on at the day of eclosion; median life span 50 days; grey: always turned off; median life span 34 days; yellow: turned off at day 10; median life span 42 days; blue: turned off at day 20; median life span 45 days), with a greater effect on life span extension shortening when turned off earlier. (C) The age specific mortality rates of shifted flies revert to the shape of the control curves (either continuously-on for the turn-on experiments, or continuously-off for the turn-off experiments) approximately 15-20 days after the food switch was executed (colors as in A and B; the day of the food switch is indicated by a black arrow, the approximate day the mortality rates have reverted is indicated by colored arrows; for statistical analysis of survivorship curves please refer to Table 1).

In order to better understand the dynamics of DN-Dmp53-dependent-life span extension we made use of the inducible GeneSwitch System for conditional expression of the DN-Dmp53 constructs in the adult fly brain. Using this system, we switched flies from food containing RU486 to non-RU food, and vice-versa. When expression of the DN-Dmp53 constructs was induced from the day of eclosion, a 47% median life span extension was observed, which is consistent with earlier observations [8]. When expression was induced later, at 10 or 20 days of adult life, median life span was still extended, but to a smaller extent (29% and 12%, respectively, Figure 1A; for statistical analysis of all life span experiments please refer to Table 1). We then performed the reverse experiment by switching flies back to non-RU486 containing food to stop induction of DN-Dmp53. These flies also showed extended life span, but the extension was again smaller than life span extension of flies with continuous expression of DN-Dmp53 (Figure 1B). While it is relatively easy to visualize the change in survivorship for flies switched at day 10, the differences for flies switched at day 20 was more subtle (Table 1). To further examine whether DN-Dmp53 expression turning on or off at day 20 has plastic, reversible effects on mortality, we compared age-specific mortality trajectories between cohorts. These data show that within 15-20 days after addition or removal of RU486 to day 20 flies there is a complete shift from the pre-switch mortality curve to the post-switch mortality curve. When plotting age-specific mortality rates (*μ_x_*) on a natural log scale, we observed that mortality trajectories of flies shifted from control food (EtOH solvent control) to food containing RU486 (expression turned on at day 20), or shifted from RU486 food to control food (expression turned off at day 20), converged and fully reverted to the mortality levels of flies constitutively held on RU486 food ("on") or on control food ("off"), respectively (Figure 1C). We used Cox (proportional hazards) regression to analyze censored mortality data post-switch and to avoid making assumptions about the particular distribution (and thus shape) of mortality rates [9]. Twenty days after the switch, mortality trajectories of shifted and non-shifted flies converged fully and became indistinguishable from each other for both "on" and "off" treatments (day 20, switch off: *p* = 0.67; day 20, switch on: *p* = 0.16). Moreover, mortality trajectories of "off" (constitutively off and switched off) versus "on" (constitutively on and switched on) cohorts differed significantly (*p* = 0.0162) from each other (excluding the last 10 days of life), confirming convergence and the effectiveness of the "on" and "off" treatments.  The delay in convergence most likely reflects some latency in induction kinetics of the RU-dependent GeneSwitch system (as well as the stop of induction), which we have also observed using a lacZ-reporter (data not shown). This reversible shift in mortality rates is reminiscent of what has been observed in flies switched from high to low calorie food (and vice-versa; data not shown and [9,10]), further supporting the idea that DN-Dmp53 and CR might employ similar mechanisms to extend life span [8,11].

**Table 1.** The effect of various interventions on female life span. Log rank analysis of the survivorship curves of female ELAV-Switch-DN-Dmp53 or dSir2 flies raised on normal food (except where indicated). Mean, median and maximum lifespan, log rank analysis, p-value, percent change in mean, median and maximum lifespan as compared to controls (without RU486 for GeneSwitch experiments), Chi-square and p-values derived from the survivorship curves for each indicated intervention are shown. Maximum life span was calculated as the median life span of the longest surviving 10% of the population. Experiments shown are representatives of at least two independent experiments.

**Intervention**	**Mean LS (vs.ctrl)**	**Mean LS extension**	**Median LS (vs. ctrl)**	**Median LS extension**	**Max LS (vs. ctrl)**	**Max LS extension**	**Number of flies (control; experimental)**	**χ2**	**p-value**
DN-Dmp53 from day 0	49/37	32%	50/34	47%	72/66	9%	267 261	48.48	<0.0001
DN-Dmp53 from day 10	46/37	24%	44/34	29%	74/66	12%	267 267	29.25	<0.0001
DN-Dmp53 from day 20	40/37	8%	38/34	12%	68/66	3%	267 247	1.883	0.17
DN-Dmp53 until day 10	43/37	16%	42/34	24%	68/66	3%	267 261	6.741	0.0094
DN-Dmp53 until day 20	46/37	24%	45/34	32%	68/66	3%	267 278	20.14	<0.0001
dSir2 (normal RU486)	50/47	6%	50/44	14%	83/80	4%	262 268	5.849	0.0156
dSir2 (normal RU486, 1.5N)	55/49	12%	56/51	10%	74/68	9%	244 247	35.05	<0.0001
dSir2 (high RU486)	56/45	24%	58/42	38%	82/76	8%	267 272	23.45	<0.0001
dSir2 (high RU486)	51/42	21%	50/38	32%	80/72	11%	251 258	20.48	<0.0001
DN-Dmp53(1.5N)	58/50	16%	60/54	11%	73/66	11%	207 272	60.36	<0.0001
dSir2 + DN-Dmp53(1.5N)	53/47	13%	55/50	10%	78/68	15%	195 204	20.22	<0.0001
Resveratrol(1.5N)	57/50	14%	58/54	7%	78/66	18%	207 276	45.07	<0.0001
Resveratrol + DN-Dmp53 (1.5N)	55/50	10%	56/54	4%	76/66	15%	207 282	28.91	<0.0001

### Life span extension by DN-Dmp53 and dSir2 are not additive.

We have previously shown that life span extension by DN-Dmp53 expression is not additive to life span extension by CR treatment [8,11]. In fact, when a variety of different food types with different calorie content are tested and mean life span is plotted against calorie content, the resulting curve of the DN-Dmp53 expressing flies is shifted towards higher calorie content (Supplementary Figure 1 and data not shown). Together with the data from [8,11], this suggests that DN-Dmp53 is part of a CR life span extending pathway. In the fly, CR is also mediated at least in part by dSir2 [7]. Since the life span extending effects of DN-Dmp53 and CR, and of CR and dSir2 are not additive, we investigated whether DN-Dmp53 and dSir2 might be part of the same life span extending pathway by determining if life span extension induced by DN-Dmp53 and dSir2 expression were also not additive.

**Figure 2.**
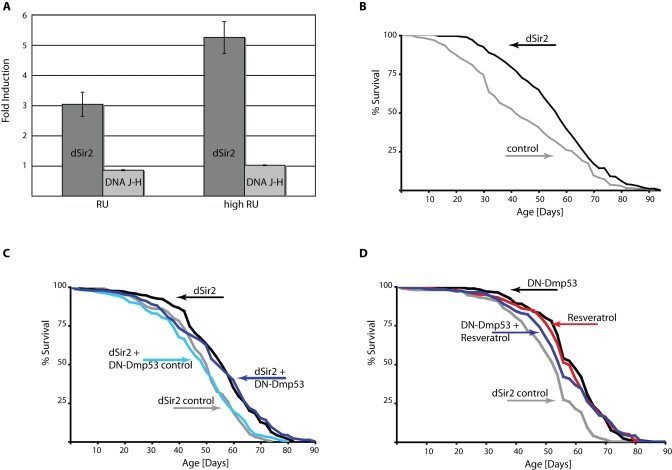
DN-Dmp53-dependent life span extension is not additive to life span extension caused by dSir2 activation. **(A)** Quantitative PCR analysis of gene induction dynamics in ELAV-Switch-EP2300 flies. Flies were raised on food containing two different doses of RU486 and harvested at day 10 of adult life. Induction of transcripts for the two genes affected by the P-element insertion (dark grey: dSir2; light grey: DNA J-H) compared to flies raised on control food were analyzed. Shown is a representative of three independent experiments (p=0.0037 for comparison of the dSir2 mRNA levels between normal and high RU doses). **(B)** Survivorship curves of female flies expressing dSir2 due to high dose RU486 treatment (grey: control; black: dSir2) show increased median life span extension of 38% (compare to [6], Table 1). **(C)** Survivorship curves of female ELAV-Switch flies expressing dSir2 alone or together with DN-Dmp53 raised on 1.5N food. Flies expressing dSir2 alone on normal RU486 conditions have median life span extended by 10% (grey: control; median life span 51 days; black dSir2; median life span 56 days). Flies additionally expressing DN-Dmp53 have an extended median life span of 10% (light blue: control; median life span 50 days; dark blue: dSir2 + DN-Dmp53; median life span 55 days), which is similar to the effects of dSir2 expression alone.**(D)** Survivorship curves of female ELAV-Switch flies expressing DN-Dmp53 with or without resveratrol treatment raised on 1.5N food. DN-Dmp53 expression alone extends median life span by 11% (grey: control; median life span 54 days; black: DN-Dmp53; median life span 60 days). Treatment of control flies with 200μM resveratrol extends median life span by 7% (red; median life span 58 days). When the two treatments are combined, median life span is extended by 4% (blue; median life span 56 days), revealing no additive effects on life span (shown are representative experiments (a selection of repetitions is shown in supplementary Figure 2); for statistical analysis of survivorship curves please refer to Table 1).

The dSir2 line EP2300 used for these experiments contains a UAS-sequences carrying P-element that is inserted in the dSir2 5'UTR. This permits the over expression of the normal dSir2 gene, including its complement of introns that may be important for efficient transcription and translation. The region, in which this P-element is inserted, however, is shared by the DNA J-H gene, whose transcriptome is arranged in the opposite direction. In order to verify that life span extension by EP2300 is indeed due to dSir2 over expression, we performed QPCR analysis of dSir2 over expressing flies. Under normal RU conditions used for life span experiments, dSir2 was ~3fold up regulated, while DNA J-H was barely changed (Figure 2A). When the RU concentration was increased, dSir2 mRNA expression was accordingly increased (~ 5fold), while DNA J-H levels remained unchanged (Figure 2A). Increasing the RU concentration in the food further extends the life span of dSir2 expressing flies (Figure 2B and Table 1), compared to flies raised on normal RU486 concentrations. These data indicate that life span extension in the EP2300 line is indeed related to a selective increase in dSir2 expression.

Next, we examined if DN-Dmp53 life span extension was additive to life span extension by dSir2. As shown in Figure 2C, no additive effects were observed when both proteins were over expressed in the adult nervous system, suggesting that the life span extending effects of DN-Dmp53 and dSir2 expression may be part of a common pathway related to CR.

### Life span extension by DN-Dmp53 and resveratrol are not additive.

Another method to activate Sir2 is by using small molecule activators of Sir2, such as resveratrol [12]. Resveratrol has been shown to extend life span of flies, yeast and worms [13,14] utilizing a pathway similar to CR [6]. Importantly, in flies life span extension by resveratrol treatment is abolished in dSir2 null flies. These data suggest that resveratrol acts to extend life span in the flies through a dSir2-dependent mechanism. Therefore, we tested whether life span extension by DN-Dmp53 and this dSir2 activator were additive. As shown in Figure 2D, life span extensions by these two different treatments were not additive. Taken together, these results suggest that CR, dSir2 and DN-Dmp53 share similar mechanisms of life span extension.

### dSir2 interacts with Dmp53 and deacetylates Dmp53-derived peptides.

**Figure 3.**
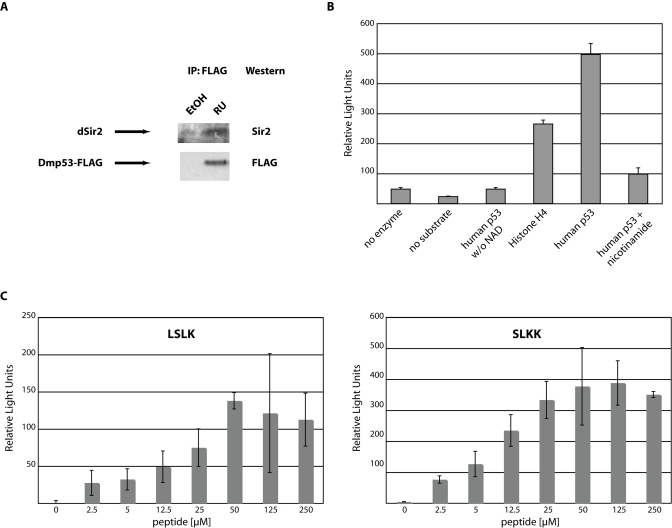
Functional interaction between dSir2 and Dmp53. **(A)** Endogenous dSir2 physically interacts with Dmp53. A FLAG-tagged version of wild type Dmp53 was expressed in females during the first 10 days of adult life using the ELAV-Switch driver. Head extracts were then immunoprecipitated with anti-FLAG antibody. Western blot analysis with an antibody against dSir2 shows efficient co-immunoprecipitation of endogenous dSir2 with the over expressed wild type Dmp53-FLAG construct. **(B)** Recombinant dSir2 deacetylates human substrates. Recombinant purified dSir2 was incubated with the indicated substrates (5μM) in triplicate and released fluorescence was measured as Relative Light Units. No deacetylation activity was observed when no NAD was added or the Sir2 inhibitor nicotinamide was added. Shown is a representative of at least three independent experiments. **(C)** Recombinant dSir2 deacetylates Dmp53-derived peptides. Recombinant purified dSir2 was incubated in triplicate with the indicated Dmp53-derived peptides. Deacetylation activity is dose-dependent and reaches saturation at higher substrate concentrations. The SLKK substrate gets deacetylated with similar efficiency as the human p53 peptide and about twice as efficiently as the LSLK peptide, suggesting substrate specificity of dSir2. The experiments shown are background corrected for non-NAD containing reactions. Shown are representatives of at least three independent experiments.

In mammalian systems Sir2 and p53 have been shown to physically interact with each other [15,16,17] ). This interaction leads to inhibition of p53 transcriptional activity. If dSir2 and Dmp53 indeed act together as a part of a CR-related pathway of life span extension in *D. melanogaster*, the observed interaction between the mammalian proteins may also occur with the fly homologs. To test this hypothesis, we expressed FLAG-tagged wild type Dmp53 in the adult nervous system using the GeneSwitch system, as expression of wild type-Dmp53 during development is lethal [8]. After induction of wt-Dmp53 expression for ten days, heads were isolated and proteins extracted. Tagged Dmp53 was immunoprecipitated with a FLAG-antibody. As can be seen in Figure 3A, endogenous dSir2 efficiently co-immunoprecipitated with over expressed FLAG-Dmp53, indicating that, as with their mammalian counterparts, dSir2 and Dmp53 physically interact.

In mammals, a consequence of the interaction between Sir2 and p53 is the deacetylation of p53 [15,16,17]. We therefore tested whether dSir2 is able to deacetylate acetylated peptides derived from human p53. Recombinant dSir2 was incubated as described [12] with human peptides from either p53 or histone H4. Both peptides were efficiently deacetylated in a NAD-dependent reaction by dSir2. These reactions were inhibited by the addition of nicotinamide (Figure 3B). Next, we tested whether dSir2 is able to deacetylate peptides that were derived from Dmp53. Peptides were tested, which contain lysine residues that are conserved between the mouse, human and fly version of p53. These peptides (LSLK and SLKK) were efficiently deacetylated in a NAD- and dose-dependent manner by dSir2 (Figure 3C). The dose response curves exhibited saturation kinetics, indicating that deacetylation is due to dSir2, not caused by unrelated hydrolysis of the acetyl-group.

Finally, we tested the functional consequences of dSir2 activation on Dmp53. We thus transfected wt-Dmp53 into *Drosophila* S2 cells together with a p53-luciferase transcriptional reporter construct. The cells were then treated with the Sir2-activator resveratrol. Resveratrol inhibited Dmp53 transcriptional activity in a dose-dependent fashion as evidenced by reduction of p53-induced luciferase activity (Supplementary Figure 2). Taken together these data indicate that, in flies, just like in mammals, Dmp53 and dSir2 functionally interact.

## Discussion

When expressed in the adult nervous system of the fruit fly, DN-Dmp53 initiates signaling events leading to extended life span. These events remain plastic, as expression of DN-Dmp53 even later in life leads to extended life spans and a corresponding shift in the mortality rate trajectory (Figure 1). Our previous data showed that life span extension through expression of DN-Dmp53 is not additive to the life span extending effects of CR [8,11]. This suggests that the events triggered by DN-Dmp53 are mechanistically related to CR. Here we further explore the relationship between CR and Dmp53.

We have previously demonstrated that the life span extending effects of CR are partially mediated by dSir2 [7]. Over expression of dSir2, like expression of DN-Dmp53, extends life span when expressed in the adult nervous system. CR treatment of dSir2 null flies does not lead to life span extension [7]. When dSir2 and DN-Dmp53 are expressed together, no additive effects on life span are observed (Figure 2). When dSir2 is activated through the use of resveratrol, similar results are observed. In support of our hypothesis, it has recently been shown in *C. elegans* that reduction of cep-1 activity (the *C. elegans* p53 homolog) extends life span; this life span extension is not additive to the life span extending effects of Sir2.1 over expression [18]. Our data also shows that, as in mammalian systems [15,16,17], in flies dSir2 and Dmp53 physically interact and dSir2 can efficiently deacetylate Dmp53-derived peptides (Figure 3). This deacetylation event leads to inhibition of Dmp53 transcriptional activity. These data indicate that Dmp53 and dSir2 share a similar life span extending mechanism. Unfortunately, we cannot answer the question of Dmp53 acetylation in response to CR conditions, as we are unable to measure Dmp53 acetylation status *in vivo* with the currently available reagents.

**Figure 4.**
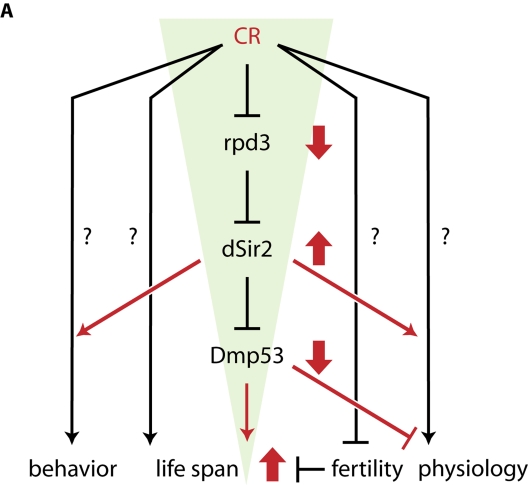
A framework for CR-dependent life span extension in *D. melanogaster*. CR treatment of flies leads to a funnel-effect: CR is a highly pleiotropic process that influences a variety of biological processes, including physiology, fertility, behavior and life span; the nature of most of these pathways remains unknown. Under CR conditions (red), rpd3 is down- and dSir2 is up regulated. dSir2 activity inhibits Dmp53 (amongst other targets), leading to life span extension. The more up stream a gene is in this pathway, the more likely it will mediate more of the pleiotropic aspects of CR, while more down stream genes only mediate some aspects of the effects of CR. For example, fertility is unchanged in dSir2- and DN-Dmp53 long-lived flies. Genetic pathways affected by all three conditions (CR, dSir2, DN-Dmp53) could be promising candidates for pathways directly influencing fly life span.

We propose a model for the life span extending effects of CR in flies (Figure 4). Our data indicate that there may be a dSir2-dependent and a dSir2-independent CR life span extension pathway, as CR increases life span to a greater extent than dSir2 or DN-Dmp53 expression by itself. The dSir2-dependent pathway is activated when, under CR conditions, activity of the histone deacetylase Rpd3 is decreased. This in turn leads to an increase in dSir2 activity by an as yet unknown mechanism [7]. Increased dSir2 activity results in increased deacetylation of Dmp53 and subsequent inhibition of its transcriptional activity. It is also possible that Dmp53 transcriptional activity, rather than being inhibited, is changed to a different set of target genes. How this change of Dmp53 activity can extend life span is currently unclear, but our recent results demonstrate that flies with decreased Dmp53 activity have lower activity of the insulin/insulin-like growth factor signaling pathway (IIS) in the fat body [11]. Reduction of IIS activity, either through the use of mutants [19] or fat body-specific dFoxO over expression [20,21] results in flies with extended life spans. It is thus conceivable that Dmp53-mediated down regulation of IIS activity might account for the some of the life span extending effects of CR. However, the activity of IIS is not limited to the regulation of dFoxO. IIS leads to the activation of the protein kinase Akt, which regulates pleiotropic signaling outputs, including activation of the transcription factor FoxO (via direct FoxO phosphorylation) or the translation initiation controlling factor 4E-BP (via phosphorylation of components of the target of rapamycin (TOR) signaling pathway).  Both of these branches are capable of controlling life span [20,21,22]. Which elements of IIS are ultimately responsible for the observed life span extending effects of DN-Dmp53 is under investigation, especially in light of the fact that life span extension due to yeast extract restriction appears independent of dFoxO [23,24].

Our data describing the association of Rpd3, Sir2 and p53 in the life span extending effects of CR provides a useful molecular genetic model that should be valuable in designing future experiments for the identification of the molecular and cellular effectors of CR life span extension.

## Experimental procedures

Fly culture and strains. All flies were kept in a humidified, temperature-controlled incubator with 12 hour on/off light cycle at 25°C in vials containing standard cornmeal medium. The ELAV-GeneSwitch line was from H. Keshishian (Yale University, New Haven, CT); UAS-Dmp53-FLAG-myc was from M. Brodsky (University of Massachusetts, Worcester, MA). UAS-Dmp53-Ct, UAS-Dmp53-259H and dSir2^EP2300^ were from the *Drosophila* Stock Center (Bloomington, IN).

Life span analysis. Flies were collected under light anesthesia, randomly divided into treatment groups and housed at a density of 25 males and 25 females each per vial. At least ten such vials were used per treatment as per [8]. Flies were passed every other day and the number of dead flies recorded.

All life span experiments were performed on regular cornmeal food (2% yeast, 10% sucrose, 5% cornmeal (all w/v)) without added live yeast, except where indicated. High and low calorie food contained varying amounts of each yeast and sucrose (w/v; from 0.2N (2%) to 1.5N (15%)), but no cornmeal [9]. For induction with the GeneSwitch system, RU486 (Sigma) was added directly to the food to a final concentration of 200μM. For experiments using high RU486 concentrations, RU486 was used at 500μM. The same concentration of solvent was added to control food. For food-switch experiments, flies were raised on RU or non-RU food, respectively, and switched to the new food regimen at the ages indicated.

Quantitative PCR. Total RNA was isolated from at least 75 heads of 10-day old females using Trizol (Invitrogen) and further purified using the RNeasy kit (Qiagen). cDNA was generated with 0.5μg total RNA using the iScript cDNA synthesis kit (Bio-Rad) in a 10μl reaction volume. 0.8μl of the iScript reaction was used as QPCR template. QPCR was performed on an ABI 7500 Real-Time PCR machine using the ABI SYBR-Green PCR master mix following the manufacturers instructions. Each QPCR reaction was performed using four biological replicates in triplicate each. The following primers were used: GAPDH-F: GAC GAA ATC AAG GCT AAG GTC G; GAPDH-R: AAT GGG TGT CGC TGA AGA AGT C; dSir2-F: TCA TCA AAA TGC TGG AGA CCA AGG; dSir2-R: TTA CTC GCT GAA TGC CTG CCA C; DNA J-H-F: ATA CGA CCT GTC CGA CTT GCG ATG; DNA J-H-R: TTC TGC TCT ACG AAA CCA CTG CCC

**Immunoprecipitation and Western Blot analysis.** UAS-Dmp53-FLAG-myc was expressed in the heads of adult flies for ten days using the ELAV-GeneSwitch driver. Approximately 75 heads per condition were isolated and homogenized in NP-40 lysis buffer (1% NP-40, 20mM Tris pH 8.0, 137mM NaCl, 2mM EDTA, 10% glycerol) plus protease inhibitors (CompleteMini, Roche). 500μg protein extracts were incubated with 1μl anti-FLAG M2 antibody (Sigma). After overnight incubation at 4°C, extracts were precipitated with 25μl Protein G-Sepharose beads (Sigma) for 30 minutes. Immunoprecipitations were resolved on a 12% SDS-PAGE and transferred to nitrocellulose membranes (BioRad). Western blots were performed with anti-FLAG M2 antibody (Sigma). Recombinant full-length dSir2 was used to immunize New Zealand White rabbits using Covance's standard 118-day protocol (Covance Research Products, Denver, PA). Polyclonal antibodies to dSir2 were purified by affinity purification from serum using the Aminolink kit (Pierce) coupled to recombinant dSir2 protein as an affinity matrix. Specificity of the antibody was verified by Western blot against wild-type and Sir2 null fly extracts.

Deacetylation Assays. Recombinant dSir2 was produced as described [14]. In short, His_6_-tagged recombinant dSir2 was purified from *E. coli* BL21(DE3) *plysS* cells harboring the pRSETc-dSir2 plasmid (gift of S. Parkhurst). Cells were grown in LB medium containing antibiotics at 30°C to an OD_600_ of 0.6-0.8. After addition of IPTG (1 mM), flasks were shifted to 16°C for 20 h. Cell pellets were resuspended in cold PBS buffer containing 300 mM NaCl, 0.5 mM DTT, 0.5 mM PMSF and EDTA-free protease inhibitor tablets and lysed by sonication. Ni^2+^-NTA beads were added to the clarified extract and after 1-3 hours they were loaded on a column, washed with buffer (20 volumes of 50 mM Tris. Cl pH 7.4, 200 mM NaCl, 30 mM imidazole) then eluted with the same buffer containing 250 mM imidazole.

Human p53- and human histone H4-derived peptides were obtained from Biomol. Dmp53-derived peptides were manufactured by Biomol. Deacetylation assays were performed as described [12] using 1μl of recombinant dSir2 and 500μM NAD (Sigma). Released fluorescence was measured using a 96-well plate reader (Biotek Instruments) and plotted as relative fluorescence compared to background (Relative Light Units, RLU).

Tissue culture. Schneider S2 cells were cultured in Schneider's *Drosophila* Medium plus 10% FCS, 1%Penicillin/Streptomycin, 10% L-glutamine (Invitrogen). Cells were transfected using FuGene6 (Roche) with pMT-Dmp53-GFP and stable transfectants were selected using Hygromycin (Roche). Three independently derived lines were used for reporter assays. 1x10^6^ cells of those stable transfectants were transfected with 0.2μg p53-Luc (Stratagene) or with 0.2 μg p53-Luc and 0.2μg pRL-SV40 (Promega) in triplicate. After overnight incubation, cells were induced with 500μM MgSO_4_ and indicated amounts of resveratrol for 4hrs. Lysates were either normalized for Dmp53 expression by a-GFP western blot or by measuring renilla luciferase activity. Luciferase activities were normalized on the reporter activity of Dmp53 expressing cells without drug treatment. Luciferase activity was assayed using the Dual Luciferase Assay System (Promega) per manufacturers instructions on a TD20/20 luminometer (Turner Designs).

Statistics. Log rank tests, were performed using the Prism suit of biostatistical software (GraphPad, San Diego). Maximum life span was calculated as the median life span of the longest surviving 10% of the population. Age-specific instantaneous mortality rate was estimated as ln(*m_x_*) ≅ ln(-ln[1-*D_x_*/*N_x_*]), where *D*_x_ is the number of dead flies in a given census interval [25].

We analyzed mortality rates using Cox regression (proportional hazards [26]) implemented in JMP IN 5.1. statistical software (SAS Institute [27]). For clarity, we smoothed age-specific mortality rates as (ln(*m_x_*)) using running averages over three census intervals (six days).

## Supplemental materials

Supplementary Figure 1 Effect of diet on the life span of DN-Dmp53 expressing flies. Mean life span of female control and DN-Dmp53 expressing flies is plotted against calorie content of the food used to raise the flies. Control flies display maximum life span at the 0.5N food, and shortened life spans at lower food concentrations (underfeeding/starvation) and higher food concentrations (overfeeding). The curve for DN-Dmp53 expressing flies is shifted toward higher calorie content, suggesting that these flies are already "genetically" calorie restricted.

Supplementary Figure 2 Representative repetitions of the life span experiments shown in Figures 1 and 2. **(A)** Expression of DN-Dmp53 later in life has beneficial effects on life span. Expression of DN-Dmp53 using the ELAV-Switch driver starting from the day of eclosion increases median life span by 19% (median life span control: 52 days, grey; DN-Dmp53: 62 days; p=0.001), while expressing DN-Dmp53 later in life (RU486 regimen starting at 20 days post eclosion) extends median life span by 12% (median life span day 20: 58 days, green; p=0.0252) over uninduced control flies. **(B)** Over expressing dSir2 is not additive to the life span extending effects of DN-Dmp53 expression. Flies over expressing dSir2 using the ELAV-Switch driver on regular food have a median life span increase of 14% (median life span control: 44 days, grey; dSir2: 50 days, black; p=0.0136), while flies expressing both dSir2 and DN-Dmp53 show a median life span extension of 18% over their respective control flies (median life span control: 44 days, light blue; dSir2/DN-Dmp53: 52 days, dark blue; p=0.0039). **(C)** Life span extension by resveratrol treatment is not additive to life span extension by DN-Dmp53 expression. Flies raised on resveratrol containing 1.5N food show a significant extension of median life span of 4% compared to untreated flies (median life span control: 48 days, grey; resveratrol: 50 days, red; p=0.0255). Flies additionally expressing DN-Dmp53 using the ELAV-Switch driver do not show significant life span extension beyond that observed with resveratrol treatment alone (median life span resveratrol/DN-Dmp53: 48 days, blue; p=0.6288).

Supplementary Figure 3 The Sir2 activating drug resveratrol inhibits Dmp53 transcriptional activity. *Drosophila* Schneider S2 cells stably expressing an inducible Dmp53-GFP construct were transfected in triplicate with a p53-responsive firefly luciferase reporter and a renilla luciferase for luciferase activity normalization purposes. Cells were then induced to express Dmp53-GFP and treated for 4hrs with the Sir2 activator resveratrol at the indicated doses or solvent control. All luciferase activity induction was normalized to control treated cells. Error bars represent the standard deviation; shown is a representative of at least three independent experiments.
