# Supplementary figures and images for: dSir2 and Dmp53 interact to mediate aspects of CR-dependent life span extension in D. melanogaster
Source: Aging (Albany NY). 2008 Nov 6;1(1):38–48. doi: 10.18632/aging.100001 (PMC2765060; doi:10.18632/aging.100001)

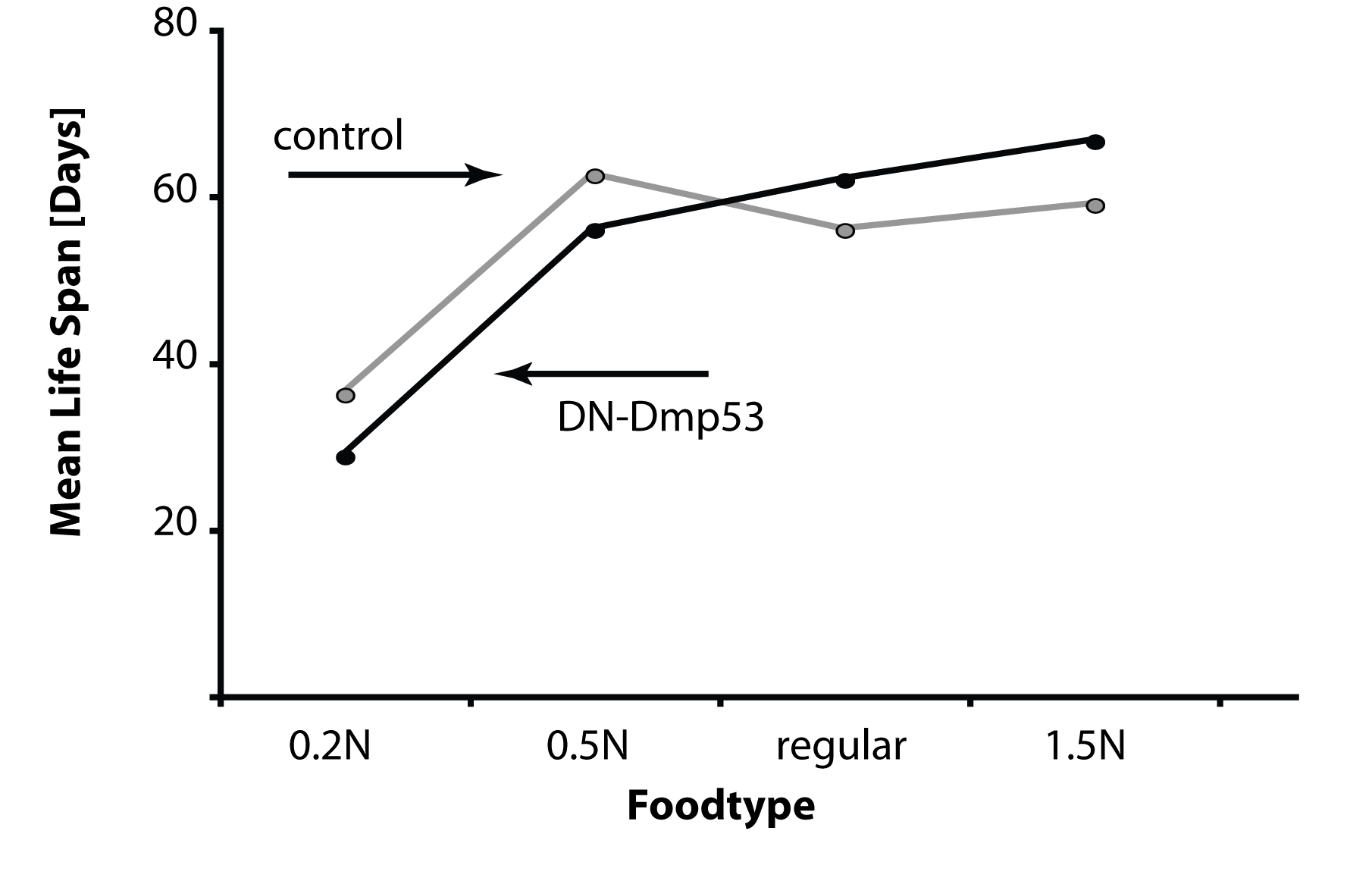

Supplement: Supplementary Figure 1 — Mean life span of female control and DN-Dmp53 expressing flies is plotted against calorie content of the food used to raise the flies. Control flies display maximum life span at the 0.5N food, and shortened life spans at lower food concentrations (underfeeding/starvation) and higher food concentrations (overfeeding). The curve for DN-Dmp53 expressing flies is shifted toward higher calorie content, suggesting that these flies are already "genetically" calorie restricted. [file aging-01-038-s001.tif]

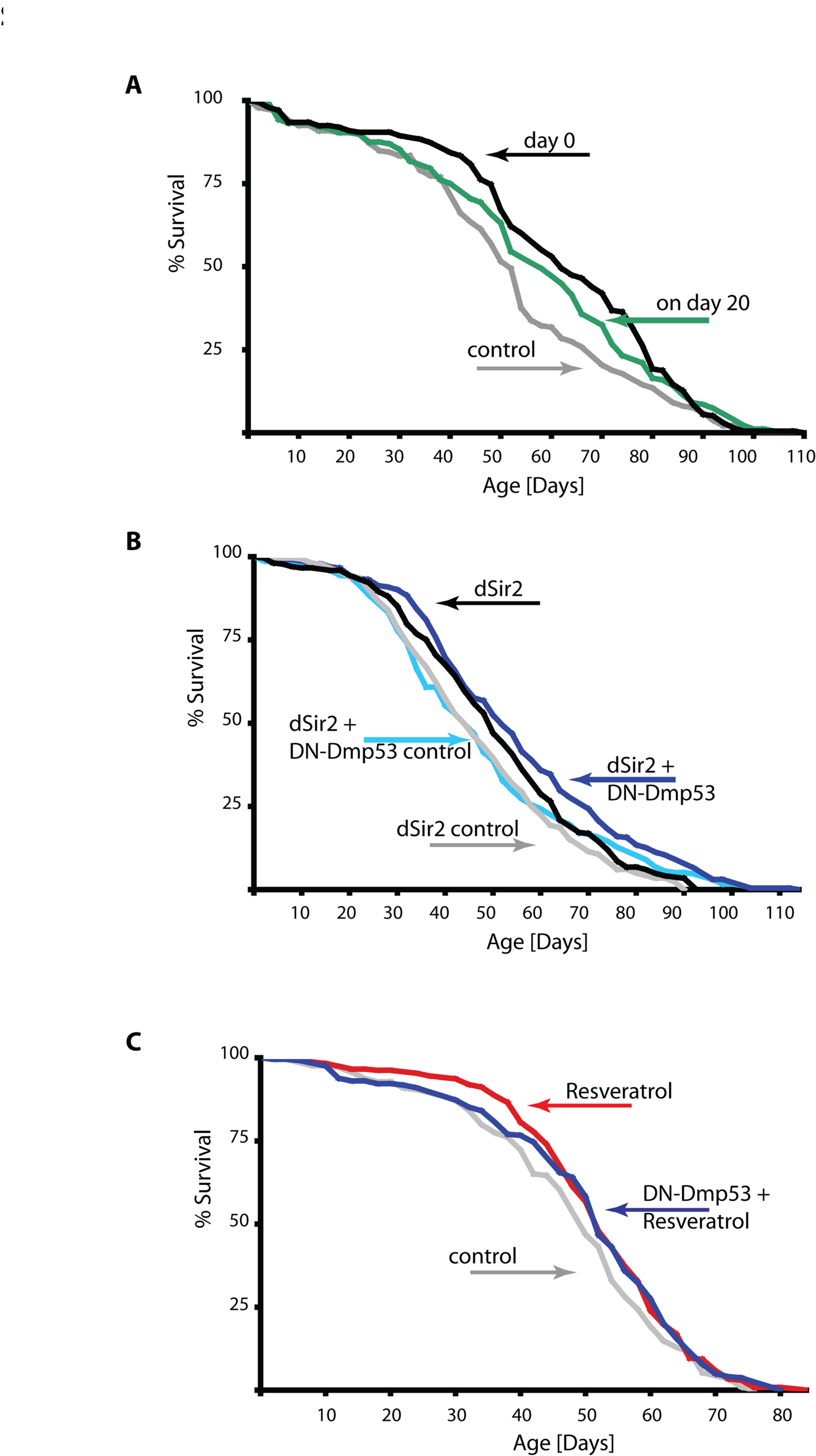

Supplement: Supplementary Figure 2 — (A) Expression of DN-Dmp53 later in life has beneficial effects on life span. Expression of DN-Dmp53 using the ELAV-Switch driver starting from the day of eclosion increases median life span by 19% (median life span control: 52 days, grey; DN-Dmp53: 62 days; p=0.001), while expressing DN-Dmp53 later in life (RU486 regimen starting at 20 days post eclosion) extends median life span by 12% (median life span day 20: 58 days, green; p=0.0252) over uninduced control flies. (B) Over expressing dSir2 is not additive to the life span extending effects of DN-Dmp53 expression. Flies over expressing dSir2 using the ELAV-Switch driver on regular food have a median life span increase of 14% (median life span control: 44 days, grey; dSir2: 50 days, black; p=0.0136), while flies expressing both dSir2 and DN-Dmp53 show a median life span extension of 18% over their respective control flies (median life span control: 44 days, light blue; dSir2/DN-Dmp53: 52 days, dark blue; p=0.0039). (C) Life span extension by resveratrol treatment is not additive to life span extension by DN-Dmp53 expression. Flies raised on resveratrol containing 1.5N food show a significant extension of median life span of 4% compared to untreated flies (median life span control: 48 days, grey; resveratrol: 50 days, red; p=0.0255). Flies additionally expressing DN-Dmp53 using the ELAV-Switch driver do not show significant life span extension beyond that observed with resveratrol treatment alone (median life span resveratrol/DN-Dmp53: 48 days, blue; p=0.6288). [file aging-01-038-s002.tif]

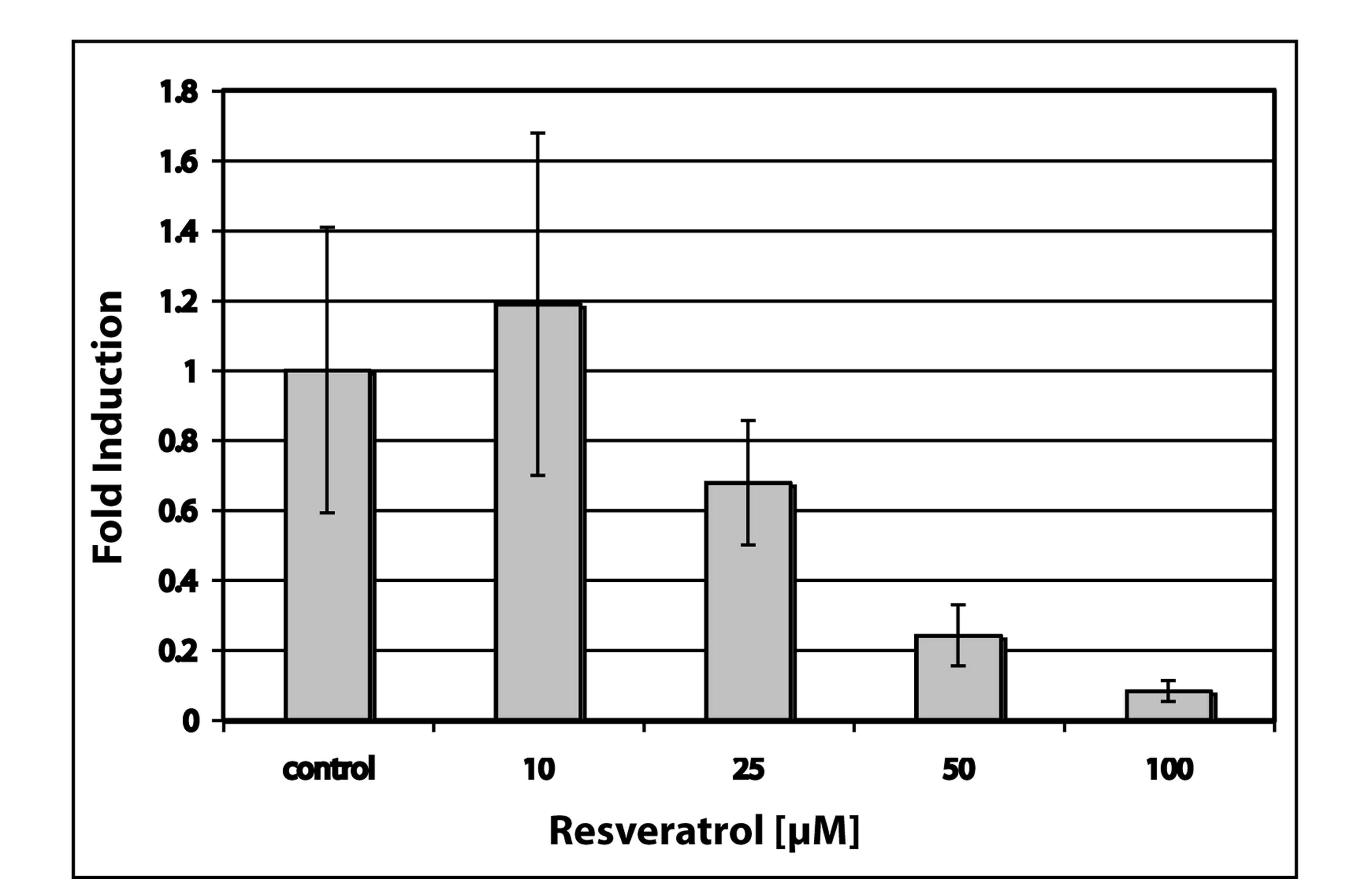

Supplement: Supplementary Figure 3 — Drosophila Schneider S2 cells stably expressing an inducible Dmp53-GFP construct were transfected in triplicate with a p53-responsive firefly luciferase reporter and a renilla luciferase for luciferase activity normalization purposes. Cells were then induced to express Dmp53-GFP and treated for 4hrs with the Sir2 activator resveratrol at the indicated doses or solvent control. All luciferase activity induction was normalized to control treated cells. Error bars represent the standard deviation; shown is a representative of at least three independent experiments. [file aging-01-038-s003.tif]
